# Telitacicept administration improved immunoglobulin A nephropathy after hematopoietic stem cell transplantation: a case report

**DOI:** 10.3389/fmed.2025.1667519

**Published:** 2025-09-15

**Authors:** Zishu Yuan, Dan Dong, Li Zhang, Zhonggao Xu

**Affiliations:** Department of Nephrology, First Hospital of Jilin University, Changchun, China

**Keywords:** IgA nephropathy, telitacicept, hematopoietic stem cell transplantation, glucocorticoids, acute myeloid leukemia

## Abstract

**Background:**

Telitacicept is a biologic that specifically targets B-lymphocyte stimulator and a proliferation-inducing ligand, demonstrating significant potential for therapeutic applications in B-cell-mediated autoimmune diseases. Currently, telitacicept is primarily utilized in the treatment of systemic lupus erythematosus. However, its therapeutic effect on immunoglobulin A nephropathy (IgAN) after hematopoietic stem cell transplantation (HSCT) has not been reported. This case report presents the clinical outcome of telitacicept administration in the treatment of IgAN following HSCT.

**Case report:**

A 36-year-old Asian man developed IgAN following allogeneic HSCT for acute myeloid leukemia (AML). The patient presented with features of high-risk nephrotic syndrome, including a urinary protein quantification of 8.28 g/24 h and serum albumin level of 29 g/L. The patient showed significant clinical improvement following treatment with moderate-dose glucocorticoids combined with telitacicept. After the treatment process, the urine albumin quantification decreased to 0.23 g/24 h, and serum albumin increased to 45.8 g/L. During the treatment, estimated glomerular filtration rate (eGFR) increased from 67.3 to 79.83 mL/min/1.73m^2^, and immune indicators immunoglobulin A (IgA), immunoglobulin G (IgG) and immunoglobulin M (IgM) also demonstrated steady levels (IgA: 6.78–10.1 g/L, IgG: 0.5–1.41 g/L, IgM: 0.17–0.6 g/L). Notably, the patient’s condition remained stable without any significant adverse effects throughout the rapid tapering of the glucocorticoid dose.

**Conclusion:**

This case suggests that telitacicept may be an effective treatment option for IgAN following HSCT, providing valuable insights into future therapeutic strategies for managing post-HSCT IgAN.

## 1 Introduction

Immunoglobulin A nephropathy (IgAN) is an immune-mediated glomerular disease and is the most prevalent form of primary glomerulonephritis in children and adults globally (1). Studies suggest that up to 20–40% of adult patients may develop renal failure, necessitating long-term renal replacement treatment (2–4). Hematopoietic stem cell transplantation (HSCT) is the principal therapy for numerous malignant and nonmalignant hematologic disorders (5). After HSCT, patients may experience adverse renal reactions, predominantly nephrotic syndrome, thrombotic microangiopathy (TMA), and calcineurin toxicity, which can evolve into chronic kidney disease (6). Minimal change disease (MCD) and membranous nephropathy (MN) are reportedly the most widespread glomerular diseases following HSCT, with IgAN also being a possible condition (7). Currently, effective guidelines for the diagnosis and treatment of IgAN after HSCT are lacking. Although glucocorticoid therapy is effective in these patients, its use has been limited due to the adverse effects of prolonged glucocorticoid administration.

Studies have revealed that IgAN following HSCT is associated with galactose-deficient IgA1 (Gd-IgA1)-mediated glomerular deposition (8). Telitacicept is a human TACIFc fusion protein that targets B-lymphocyte stimulator (BLyS) and a proliferation-inducing ligand (APRIL) and thus suppresses B-cell overexpression, resulting in a reduction of Gd-IgA1 synthesis and delay in IgAN progression. Telitacicept has been approved for the treatment of systemic lupus erythematosus in China (9). However, its potential therapeutic benefit in IgAN following HSCT has not been reported. This case report aims to present the clinical outcome of telitacicept treatment in IgAN following HSCT. We present a patient who was initially diagnosed with acute myeloid leukemia (AML) and subsequently developed IgAN after undergoing allogeneic HSCT. Notably, the patient showed marked improvement after treatment with glucocorticoids and telitacicept. The patient’s condition remained stable during the rapid tapering of glucocorticoids, which also minimized adverse effects associated with prolonged glucocorticoid use.

## 2 Case report

A 36-year-old Asian man was admitted to our hospital on December 13, 2022, with bilateral lower leg pitting edema that had persisted for over a month. Four days prior to admission, the patient experienced an unexpected elevation in blood pressure, reaching 180/120 mmHg (home blood pressure). The patient presented with a 1-year history of hypertension, with a maximum recorded blood pressure of 180/120 mmHg (home blood pressure), but had not adhered to prescribed antihypertensive medications or maintained regular blood pressure monitoring. Seven years ago, the patient was diagnosed with AML (M2 type, low-risk group) at the department of hematology of First Hospital of Jilin University. The IA regimen induction (idarubicin for 3 days and cytarabine for 7 days) achieved complete remission, followed by four courses of high-dose cytarabine consolidation therapy, which maintained complete remission and negative minimal residual disease (MRD). Four years ago, a hematological relapse was detected during routine follow-up. The patient achieved remission again with IA regimen induction and subsequently underwent allogeneic HSCT. Cyclosporine, mycophenolate mofetil, and short-course methotrexate were used for acute graft-versus-host disease (aGVHD) prophylaxis, with no occurrence of aGVHD or chronic graft-versus-host disease (cGVHD) postoperatively. Mycophenolate mofetil was discontinued after 1 month, and cyclosporine was discontinued after 7 months. During regular post-transplant follow-up, the leukemia remained in continued complete remission status with negative MRD and complete donor chimerism. The patient did not receive prophylaxis or immunization before admission.

**Figure 1:**
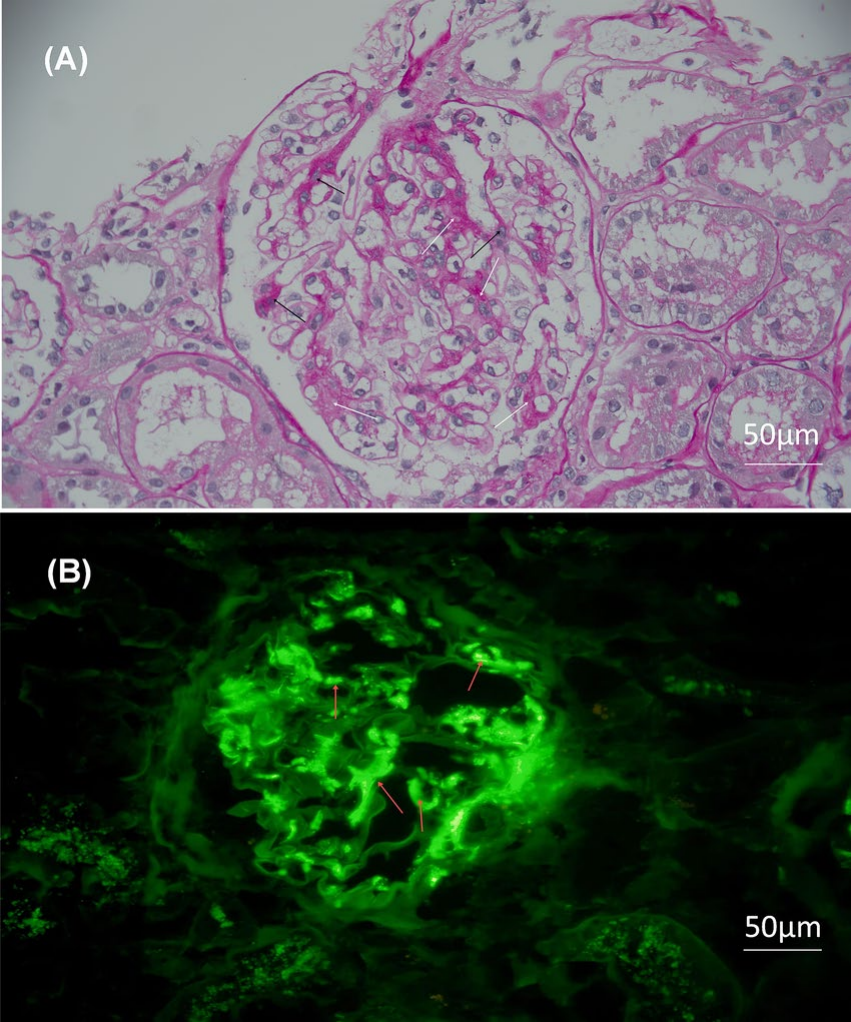
Light microscopy and immunofluorescence of the biopsy. Original magnification 200×, bar = 50 μm. **(A)** Light microscopy: periodic acid–Schiff staining reveals significant hyperplasia of mesangial cells and matrix. The white arrows represent the proliferation of the mesangial matrix, while the black arrows represent the proliferation of the mesangial cells. **(B)** Immunofluorescence: the red arrows indicate the deposition of immunoglobulin A in the mesangial area of the glomerulus in forms of clumps.

**Figure 2:**
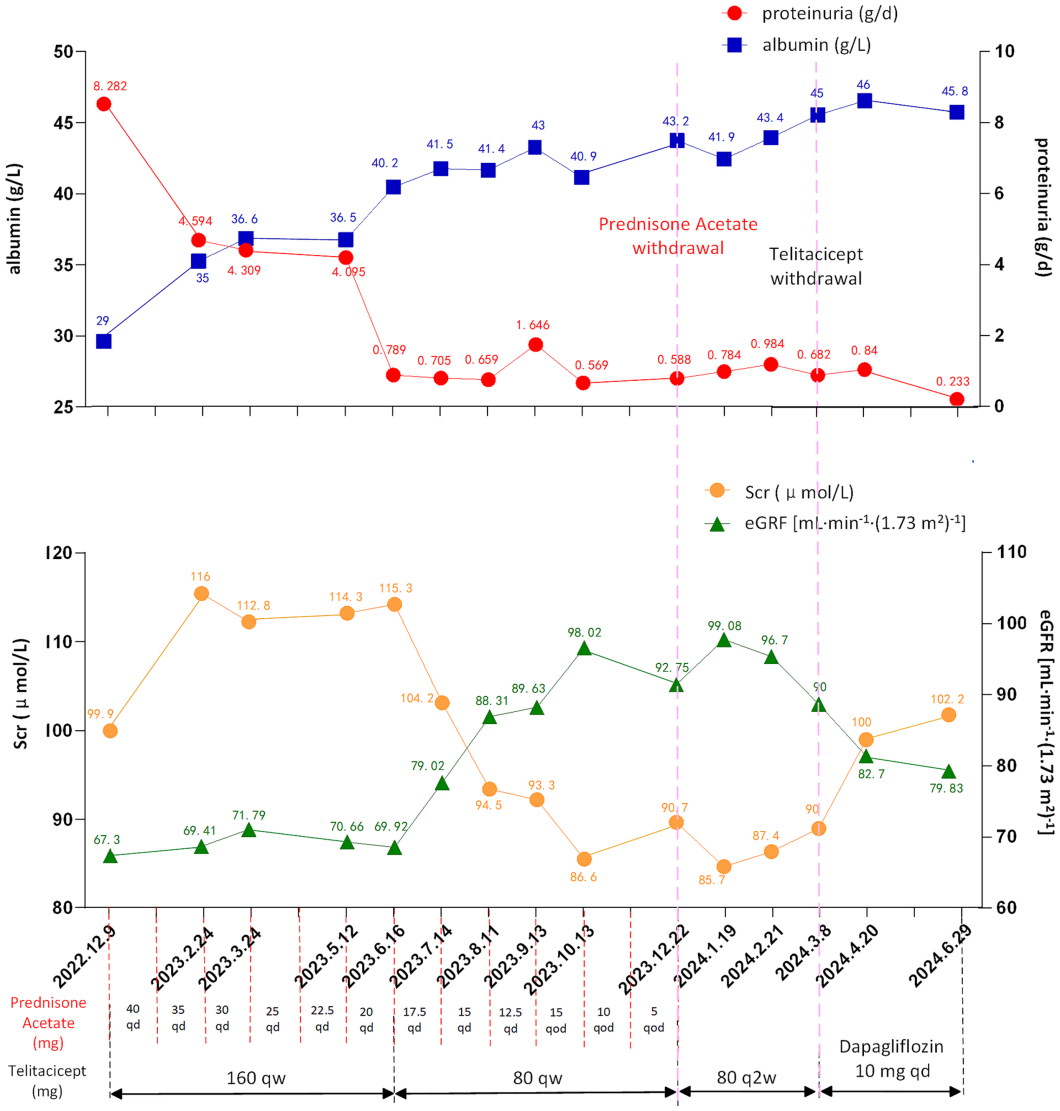
Changes of proteinuria (reference range: <0.15 g/day), albumin (reference range: 40–55 g/L), Scr (reference range: 57–97 μmol/L) and eGFR (reference range: >90 mL/min/1.73m^2^) levels during treatment. eGFR, estimated glomerular filtration rate; Scr, serum creatinine; qd, once a day; qod, every other day; qw, once a week; q2w, once every 2 weeks.

**Figure 3:**
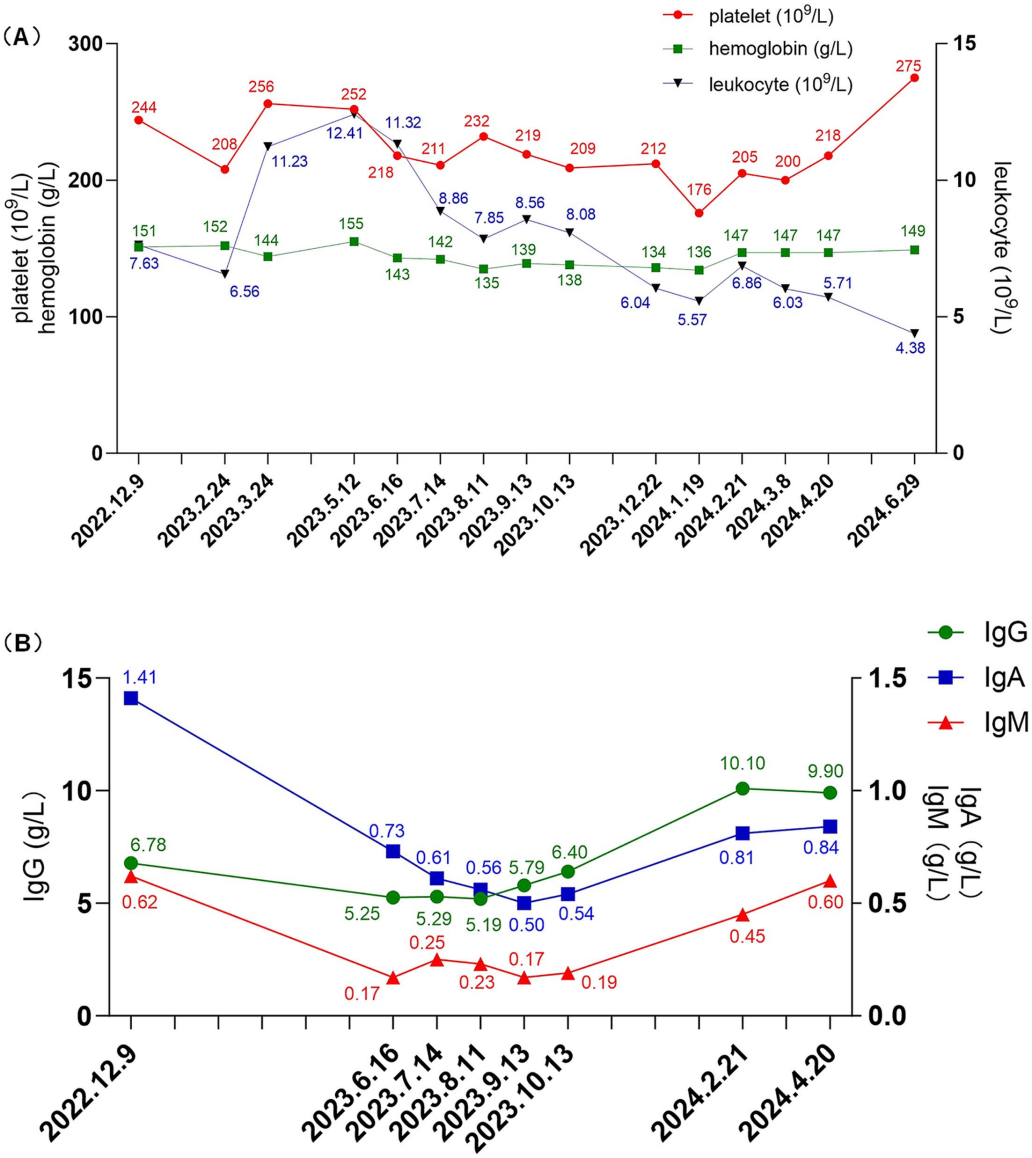
**(A)** Changes in leukocyte (reference range: 3.5–9.5 × 10^9^/L), platelet (reference range: 125–350 × 10^9^/L), and hemoglobin levels (reference range: 130–175 g/L) during treatment. **(B)** Changes in IgG (reference range: 8.6–17.4 g/L), IgA (reference range: 1.0–4.2 g/L), and IgM (reference range: 0.5–2.8 g/L) levels during treatment. IgA, immunoglobulin A; IgG, immunoglobulin G; IgM, immunoglobulin M.

The body mass index (BMI) of patient was 36.83 kg/m^2^. The laboratory and imaging examinations were also performed upon admission. The complete blood count revealed a white blood cell count of 5.08 × 10^9^/L, red blood cell count of 4.08 × 10^12^/L, hemoglobin level of 138 g/L, and platelet count of 212 × 10^9^/L. Urinalysis revealed 3+ occult blood, 3+ proteinuria, and a red blood cell count of 44.8/high-power field. The alanine aminotransferase level was 18.9 U/L. aspartate aminotransferase level was 18.3 U/L. The alkaline phosphatase level was 51.3 U/L, and gamma-glutamyl transferase level was 21 U/L. The serum albumin level was 29 g/L. Renal function tests showed a urea level of 8.91 mmol/L, creatinine level of 99.9 μmol/L, uric acid level of 451 μmol/L and estimated glomerular filtration rate (eGFR) of 67.3 mL/min/1.73m^2^. Lipid profile results showed a cholesterol level of 6.10 mmol/L, triglyceride level of 1.81 mmol/L, and low-density lipoprotein cholesterol level of 4.35 mmol/L. The 24 h (24 h) urine albumin quantification was 8.282 g. Immunologic assays indicated an immunoglobulin G (IgG) level of 6.78 g/L, IgA level of 1.41 g/L, immunoglobulin M (IgM) level of 0.62 g/L. Consequently, the patient was diagnosed with IgAN following kidney biopsy (grade III IgAN according to Lee grading and Oxford score M1E1S1T0-C0). The renal biopsy pathology results are presented in Figure 1. The treatment commenced with the administration of prednisone acetate 40 mg daily and telitacicept 160 mg weekly from December 19, 2022. Simultaneously, nifedipine 30 mg once daily and sacubitril/valsartan 24/26 mg twice daily were prescribed to control blood pressure. During follow-up, the doses of glucocorticoids and telitacicept were gradually tapered, guided by clinical judgment and according to the patient’s condition. After 6 months (June 16, 2023), the 24 h urine albumin quantification was 0.789 g, so the dosage of telitacicept was reduced to 80 mg weekly and the doses of glucocorticoids were gradually tapered. After 12 months (December 22, 2023), the 24 h urine albumin quantification was 0.588 g, so telitacicept was further reduced to 80 mg once every 2 weeks and glucocorticoids were discontinued. On March 8, 2024, the 24 h urine albumin quantification was 0.682 g, and telitacicept was discontinued. After discontinuing telitacicept, supportive therapy was optimized, with dapagliflozin 10 mg once daily. During treatment, the patient’s hematuria, proteinuria, and hypoproteinemia gradually improved, with renal function maintaining a stable trajectory, and no serious adverse events occurred. The patient maintained regular administration of nifedipine and sacubitril/valsartan at the initially prescribed doses, achieving adequate blood pressure control (120–130/70–80 mmHg), and did not experience obvious weight gain or loss during the treatment. The alterations in the 24 h urine albumin quantification level, serum albumin levels, and renal function during the disease are presented in Figure 2. Fluctuations in complete blood count indicators are illustrated in Figure 3A, and variations in IgA, IgG, and IgM levels are presented in Figure 3B.

## 3 Discussion

IgAN is typically classified into primary and secondary forms. The patient developed IgAN after HSCT, and graft-versus-host disease (GVHD) is a known significant complication associated with allogeneic HSCT. GVHD is an immune-mediated condition that affects multiple organs (including the liver, gastrointestinal tract, and skin) and occurs when donor T cells respond to incompatible antigens within the host (10). aGVHD typically manifests within the first 3 to 6 months post-transplant. However, cGVHD can emerge more than 3 months after allogeneic HSCT. cGVHD is primarily diagnosed based on clinical manifestations, resembling autoimmune diseases, and can involve one or more organs throughout the body, with the skin, hair, nails, oral cavity, liver, eyes, gastrointestinal tract, genitalia, and joints or fascia being the most commonly affected. cGVHD can also present with characteristic laboratory abnormalities, including elevated total bilirubin, thrombocytopenia, reduced peripheral blood lymphocyte and eosinophil counts, as well as alterations in gamma globulin levels, which may manifest as either a decrease or an increase (11, 12). Pathological biopsy of patients with cGVHD-induced renal injury can also present various manifestations. Among them, MCD, MN and TMA are relatively common pathological types (13–15), and it can also be manifested as tubulointerstitial lesions, double-track sign of glomerular basement membrane, segmental “spikes,” etc. (16). A diagnosis of cGVHD is confirmed when a patient who has received an allogeneic transplant presents with at least one diagnostic sign of cGVHD or at least one distinctive sign of cGVHD, supported by positive examinations (such as histopathology, laboratory tests, and pulmonary function tests) (10, 11, 17, 18). In the present case, the patient developed IgAN 4 years post-HSCT and presented with no extra renal manifestations. Therefore, the diagnosis of primary IgAN was considered instead of cGVHD. Another important reason, the deposition of Gd-IgA1 within glomeruli is the pathogenic mechanisms of primary IgAN, as outlined by the widely accepted “four-hit hypothesis” (19–21). The expression of Gd-IgA1 in the blood of patients with IgAN following HSCT significantly increases, suggesting that the onset of IgAN post-HSCT is correlated with glomerular deposition mediated by Gd-IgA1 (8). Gd-IgA1 is antigenic, and its atypical hinge region induces the production of aberrant IgG and IgA antibodies. IgG antibodies can specifically target Gd-IgA1 and form immune complexes that are deposited within the mesangial region of the glomeruli, triggering mesangial cell injury, proliferation, and accumulation of the mesangial matrix. Ultimately, this cascade leads to glomerulosclerosis and interstitial fibrosis. Additionally, the process activates the complement system, leading to the release of cytokines and growth factors, which in turn facilitate inflammation (22). In this case, the suppression of Gd-IgA1 production by telitacicept effectively reduced the patient’s albuminuria levels, indicating that the patient’s albuminuria is associated with the deposition of Gd-IgA1 in the kidneys, i.e., the patient has primary IgAN rather than cGVHD-associated nephropathy.

The possible molecular mechanisms of renal injury following HSCT is unclear. It may be due to the dysregulation of T cell subsets after HSCT, leading to enhanced autoimmune responses (23). The development of MN after HSCT may be also associated with positive IgA-κ deposits (24). FAT1 antigen is a specific marker of HSCT-related MN. Anti-FAT1 antibodies detected in the serum of patients after HSCT can directly act on the FAT1 antigen of podocytes, causing podocyte injury and the occurrence of MN (25). The development of TMA following HSCT is primarily attributed to persistent endothelial injury induced by factors such as medications, allogeneic immune reactions, infections, or antibody formation. This ongoing endothelial damage activates the complement system, ultimately leading to the formation of microthrombi within the microvasculature (26). Moreover, the immune conditioning regimen used in HSCT leads to profound B-cell depletion, followed by gradual B-cell reconstitution after allogeneic HSCT. This process may result in relative B-lymphopenia and elevated levels of BlyS (27). BLyS is an important member of the tumor necrosis factor family that primarily interacts with three receptors on the cell surface: B-cell activating factor receptor, transmembrane activator and CAML interactor (TACI) and B-cell maturation antigen (BCMA). It promotes the transition of transitional B cells into mature B cells while enhancing the proliferation and survival of these mature B cells (28, 29). APRIL and BlyS are both members of the tumor necrosis factor ligand superfamily and function synergistically within the BlyS/APRIL system. APRIL predominantly interacts with the TACI and BCMA receptors in B cells. It plays a critical role in antibody class switching and plasma cell survival, and is particularly involved in the late stages of B-cell differentiation (30). Therefore, alterations in the BlyS/APRIL system following HSCT may have significant effects on B-cell dynamics. In IgAN, elevated levels of both BlyS and APRIL can modulate B-cell signaling pathways, thereby promoting the survival of IgA-secreting plasma cells and increasing circulating levels of Gd-IgA1 antibodies (31, 32). These findings suggest that HSCT may indirectly influence the BlyS/APRIL-B-cell-Gd-IgA1 signaling axis and induce IgAN. This illustrates a potential pathogenic mechanism of IgAN following HSCT.

The patient’s urine albumin quantification was >3.5 g/24 h, accompanied by hyperlipidemia, hypoalbuminemia, and symmetrical pitting edema in both lower extremities, all of which are characteristic features of nephrotic syndrome. The patient was obese with a 36.83 kg/m^2^ BMI. After a comprehensive evaluation, treatment was initiated with a moderate dose of glucocorticoids combined with telitacicept. A corticosteroid-sparing regimen was adopted due to the patient’s obesity, compounded by their expressed concerns regarding corticosteroid-induced cosmetic changes and potential adverse effects. Notably, this case report represents the first documented use of telitacicept in a patient with IgAN following HSCT. Glucocorticoids were prescribed to suppress inflammatory responses, while telitacicept specifically targeted the inhibition of B cell proliferation and differentiation, leading to reduced Gd-IgA1 production, decreased urinary protein levels, and potential slowing of IgAN progression. Telitacicept is a fusion protein composed of the extracellular domain of the soluble BLyS/APRIL receptor and the Fc region of IgG1. Therefore, this configuration can inhibit the differentiation and proliferation of B cells, thereby suppressing the aberrant production of autoantibodies by plasma cells and reducing the synthesis of Gd-IgA1 (33, 34). The use of telitacicept resulted in the rapid remission of proteinuria, shortened treatment duration, and reduced glucocorticoid dosage, thereby minimizing steroid-associated side effects.

In this case, the patient had a history of AML, and the safety of telitacicept in the context of AML was evaluated. Throughout the treatment, the patient’s complete blood count parameters, including hemoglobin, platelet, and leukocyte, remained stable. The bone marrow aspiration and biopsy after treatment revealed no significant abnormalities and the patient sustained complete remission with negative minimal residual disease. The results indicate that treatment with prednisone acetate combined with telitacicept could be considered fort IgAN after HSCT, demonstrating both favorable efficacy and an acceptable safety profile. However, this case report presents several limitations. First, the findings are derived from a single patient who received telitacicept following HSCT. More similar cases will be needed in the future to verify the universality of the conclusion. Second, the absence of a control group receiving alternative therapies limits our ability to establish comparative efficacy. Third, the relatively short post-treatment follow-up period may not fully capture long-term outcomes or potential late-onset adverse effects, so further follow-up is still needed. Finally, the concurrent use of prednisone throughout the treatment course introduces potential confounding effects that could influence the observed clinical responses. These limitations highlight the need for future attention to the occurrence of similar cases, as well as longer follow-up durations to better characterize telitacicept’s role in IgA post-HSCT management.

## 4 Conclusion

To our knowledge, this case presents the first successful use of telitacicept in the treatment of IgAN following HSCT. It demonstrates that the combination of moderate-dose glucocorticoids and telitacicept can rapidly induce remission of IgAN following HSCT while ensuring a high level of safety. This study provides important insights and may serve as a reference for future treatment strategies to address the emergence of IgAN following HSCT.

## Patient perspective

After the treatment, my kidney function improved significantly—proteinuria and hematuria decreased without any side effects. My blood pressure stabilized, and my leukemia remains in remission. Grateful for this stable recovery.

## Data Availability

The original contributions presented in the study are included in the article/supplementary material, further inquiries can be directed to the corresponding author.
